# Rituximab, gemcitabine and oxaliplatin in relapsed or refractory indolent and mantle cell lymphoma: results of a multicenter phase I/II-study of the German Low Grade Lymphoma Study Group

**DOI:** 10.1007/s00277-024-05689-w

**Published:** 2024-03-09

**Authors:** Gabriel Scheubeck, Martin Hoffmann, Vindi Jurinovic, Luca Fischer, Michael Unterhalt, Christian Schmidt, Hans-Peter Böck, Ulrich Dührsen, Joachim Kaesberger, Stephan Kremers, Hans-Walter Lindemann, Luisa Mantovani, Wolfgang Hiddemann, Eva Hoster, Martin Dreyling, on behalf of the German Lymphoma Alliance (GLA)

**Affiliations:** 1 Department of Internal Medicine III, LMU University Hospital, LMU Munich, Munich, Germany; 2 Medical Clinic A, Clinical Centre Ludwigshafen, Ludwigshafen, Germany; 3 Institute for Medical Information Processing, Biometry, and Epidemiology, LMU Munich, Munich, Germany; 4 Hematology-Oncology practice, Offenbach, Germany; 5 Clinic of Hematology, University Hospital, University of Duisburg-Essen, Essen, Germany; 6 Internal Medicine, Diakonieklinikum, Stuttgart, Germany; 7 Hematology-Oncology, Caritas Hospital Lebach, Lebach, Germany; 8 Hematology-Oncology, Catholic Clinic, Hagen, Germany; 9 Hematology-Oncology, St. Georg Clinic, Leipzig, Germany

**Keywords:** Relapsed or refractory B-cell lymphoma, Mantle cell lymphoma, Follicular lymphoma, R-GemOx, Phase I/II trial, maximum tolerated dose

## Abstract

Rituximab, gemcitabine and oxaliplatin (R-GemOx) has demonstrated to be effective and safe in lymphoma patients. We aimed to determine the maximum tolerated dose (MTD) of oxaliplatin in combination with rituximab and gemcitabine and to explore the efficacy and safety of R-GemOx in relapsed or refractory (r/r) indolent and mantle cell lymphoma (MCL). In this single-arm, phase I/II trial, we enrolled 55 patients with r/r indolent lymphoma and MCL not suitable for autologous stem-cell transplantation. Patients received 4 cycles of R-GemOx. In the dose escalation group, 70 mg/m^2^ of oxaliplatin was applied and interindividually increased by 10 mg/m^2^ until the MTD was reached together with fixed doses of rituximab and gemcitabine. At the oxaliplatin MTD, an extension cohort was opened. Primary aim was to detect an overall response rate (ORR) greater than 65% (α = 0.05). Oxaliplatin 70 mg/m^2^ (MTD) was chosen for the extension cohort after 3 of 6 patients experienced a DLT at 80 mg/m^2^. Among 46 patients evaluable for the efficacy analysis ORR was 72% (33/46), missing the primary aim of the study (*p* = 0.21). After a median follow-up of 7.9 years, median PFS and OS were 1.0 and 2.1 years. Most frequent grade ≥ 3 adverse events were cytopenias. R-GemOx induces decent response rates in r/r indolent lymphoma and MCL, though novel targeted therapies have largely replaced chemotherapy in the relapse setting. Particularly in MCL, R-GemOx might be an alternative option in late relapses or as bridging to CAR-T-cells. This study was registered with ClinicalTrials.gov on Aug 4th, 2009, number NCT00954005.

**Supplementary Information:**

The online version contains supplementary material available at 10.1007/s00277-024-05689-w.

## Introduction

Indolent lymphoma account for approximately 35–40% of all malignant lymphoma and are generally characterized by slow progression and reccurring relapses [1]. Follicular lymphoma (FL) represents the most common subtype of indolent lymphoma. Most patients are diagnosed in advanced stage III or IV according to Ann Arbor. When requiring treatment, these patients are managed with immunochemotherapy [2–5]. The prognosis is usually favorable with an overall survival of almost two decades although advanced indolent lymphoma is not considered curable [6, 7]. On the other hand, mantle cell lymphoma (MCL) typically shows a more aggressive course of disease and has the worst prognosis among B-cell lymphoma [8]. Furthermore, early disease progression within 24 months (POD24) is associated with a poor outcome in advanced stage FL and MCL [9–12]. While conventional immunochemotherapy can be repeated in relapses after long-term remission, early relapses require alternative therapy regimens. High-dose therapy (HDT) followed by autologous stem-cell transplantation (ASCT) has been considered standard in first-line treatment for younger MCL patients [13–15]. Interestingly, recently published data from the TRIANGLE trial suggests that the addition of ibrutinib to the induction treatment might substitute ASCT in the majority of MCL patients [16]. ASCT is also effective in patients with FL [17, 18], but is only considered superior in early relapses of younger patients without comorbidities [19, 20].

Lately, new approaches such as rituximab-lenalidomide (R2) in FL or ibrutinib in MCL have expanded the therapeutic landscape in relapsed NHL [16, 21–23]. However, conventional chemotherapy still remains an option after long-term remissions. Rituximab in combination with fludarabine, cyclophosphamide and mitoxantrone (R-FCM) demonstrated a good efficacy in relapsed FL and MCL patients with an overall response rate of 79% [24]. Gemcitabine as well as oxaliplatin demonstrated a promising single-agent activity in multiple lymphoma entities [25–31]. Both substances were also capable of inducing responses in refractory disease [25, 30]. Particularly interesting is the good efficacy in MCL [25, 30]. As both substances exhibit a different side-effect profile, they seem very suitable for combination therapy. Additionally, as compared to cisplatin, oxaliplatin features an improved safety profile regarding renal toxicity making this substance highly attractive for elderly and comorbid patients [30, 32, 33]. In aggressive NHL, the combination of gemcitabine and oxaliplatin together with Rituximab (R-GemOx) showed promising activity and an acceptable toxicity profile with predominantly hematological toxicities in several phase II trials [34–36]. As CHOP- or bendamustine-based regimens are widely applied in first-line, gemcitabine and oxaliplatin represent cytostatic agents without known cross-resistance to these drugs.

Aim of this study was to evaluate a novel non-cross resistant treatment option for relapsed or refractory (r/r) indolent lymphoma and MCL. At the time of the study design, no phase I study existed which had investigated the combination of gemcitabine and oxaliplatin in indolent and mantle cell lymphoma. Based on these considerations, the German Low Grade Lymphoma Study Group (GLSG) initiated a phase I/II trial to determine the maximum tolerated dose (MTD) of oxaliplatin in combination with gemcitabine and to explore the efficacy of the R-GemOx regimen with the prespecified MTD of oxaliplatin in r/r indolent and mantle cell lymphoma patients.

## Patients and methods

### Study design and patients

The R-GO study (NCT00954005) was a prospective, single-arm, multicenter national phase I/II trial conducted at 21 centers in Germany initiated by the GLSG. R-GO was designed to explore safety, tolerability and efficacy of rituximab, gemcitabine and oxaliplatin. Eligible patients were ≥ 18 years of age and had relapsed or refractory histologically confirmed indolent B-cell non-Hodgkin’s lymphoma or MCL according to the World Health Organization (WHO) classification from 1997 [37], were in need of treatment, had Eastern Cooperative Oncology Group (ECOG) performance status of 0 to 2, an estimated life expectancy of 12 weeks or more and at least one bi-dimensionally measurable lesion. Patients must not have received cytotoxic treatment within 4 weeks before study entry. We excluded patients suitable for high-dose chemotherapy, with transformation in high grade lymphoma, HIV-infection, and hematopoietic insufficiency with leucocyte counts < 1.5 × 10^9^ cells per L or thrombocyte counts < 100 × 10^9^ cells per L unless caused by lymphoma. Key eligibility criteria are listed in the supplemental Appendix.

All procedures followed were in accordance with the ethical standards of the responsible committee on human experimentation (institutional and national) and with the Helsinki Declaration of 1975, as revised in 2008. All patients provided written informed consent.

### Treatment

The initial study design intended to treat patients with the combination of gemcitabine and oxaliplatin (GemOx). After demonstrating a significant advantage in PFS for the addition of rituximab in combination with FCM in r/r lymphoma, an amendment was implemented on 17.11.2004 [24]. From this time, all patients received rituximab-gemcitabine and oxaliplatin (R-GemOx).

The study treatment comprised four 28-day cycles of R-GemOx. Rituximab was administered at a dose of 375 mg/m^2^ by intravenous infusion on day 0 or 1. Gemcitabine was applied intravenously at a dose of 1000 mg/m^2^ on days 1 and 15.

In the phase I dose escalation cohort, oxaliplatin was given in a 3 + 3 design on days 1 and 15 by intravenous infusion, starting at a dose of 70 mg/m^2^ and increased by steps of 10 mg/m^2^ until the maximum tolerated dose (MTD) was reached. If one out of three patients from one dose level experienced a DLT, the cohort was expanded to six patients. The MTD was defined as the dose at which a further dose step led to dose-limiting toxicities (DLTs) in ≥ 2 out of three, or ≥ 2 out of six patients after expansion of the cohort.

DLT was defined as one of the following side effects in the first cycle: Neutropenia < 0.5 × 10^9^ cells per L, thrombocytopenia < 25 × 10^9^ cells per L or bleeding with thrombocytopenia, neutropenia < 1.5 × 10^9^ cells per L or thrombocytopenia < 100 × 10^9^ cells per L on day 29 of the first cycle, non-hematological toxicity WHO grade 3 with exception of alopecia and nausea or emesis, or persistence of any toxicity WHO grade ≥ 2 until day 29 of the first cycle. No intraindividual dose increases were made. In case of a DLT on day 15, treatment was delayed until the absolute neutrophil count (ANC) recovered to > 0.5 × 10^9^ cells per L, platelets to > 25 × 10^9^ cells per L or bleeding was stopped.

In the phase II cohort, oxaliplatin was intravenously applied at a fixed dose of 70 mg/m^2^ (the MTD) on days 1 and 15.

An interim staging was performed after 2 cycles. In case of complete remission (CR), partial remission (PR), objective response (OR) or stable disease (SD), treatment should be completed with another 2 cycles. In case of progressive disease (PD) or non-hematological WHO toxicity grade 4, therapy was discontinued. Cycles were postponed for a maximum of 21 days, until ANC reached ≥ 1.5 × 10^9^ cells per L and the platelet count reached ≥ 100 × 10^9^ cells per L or any non-hematological response resolved to grade ≤ 2 (except for alopecia and emesis).

Patients discontinued study treatment if the next cycle was delayed by more than 21 days. However, these patients remained evaluable for the final analysis. Dose adjustments were recommended for patients who developed febrile neutropenia and WHO grade 4 hematotoxicity, with a 25% reduction in the planned doses of oxaliplatin and gemcitabine. For non-hematological toxicity of grade 3 (grade 4 for nausea and vomiting) a dose reduction by 25%, for non-hematological toxicity of grade 4 (except for nausea and vomiting) a dose reduction by 50% was foreseen.

### Study endpoints

#### Primary endpoints

In the phase I dose escalation cohort, the primary end point was DLT of oxaliplatin in combination with gemcitabine and rituximab.

In the phase II cohort, the primary end point was overall response rate (ORR), defined by CR and PR. Patients who achieved PR or CR at the interim staging but progressed or died before the completion of the therapy were assessed as treatment failures.

#### Secondary endpoints

Secondary endpoints were safety, progression-free survival (PFS), time-to-treatment failure (TTF), overall survival (OS) and the rate of CR. Parameters were also reported in an intention-to-treat analysis of all registered patients. The efficacy analysis comprised all evaluable patients of the phase I and phase II. Three patients of the phase I that were excluded due to incorrect dosage were included in the response and survival analysis.

Progression-free survival was defined by the time of study registration until progression or death from any cause. Time-to-Treatment failure was the time from initiation of chemotherapy to its premature discontinuation. Overall survival was calculated from the time of study registration until death. The rate of complete remission was assessed after completion of study therapy.

Response to treatment was assessed by clinical examination, laboratory testing, bone marrow examinations and computed tomography scan of the neck, chest and abdomen at baseline, after 2 treatment cycles and at the end of therapy (after 4 cycles). Repeated bone marrow biopsies were only performed when lymphoma infiltration was present at baseline. Adverse events were assessed according to WHO toxicity grades (WHO Handbook for reporting results of cancer treatment, No. 48 (1979)).

### Sample size and statistical analysis

In a previous trial of the GLSG, the FCM regimen (fludarabine, cyclophosphamide and mitoxantrone) induced an ORR of 58% [46%, 71%] [24]. The combination of FCM with rituximab significantly improved the ORR to 79% [67%, 88%] in the randomized trial [24]. A remission rate of 65% or less would certainly not be adequate for a randomized comparison with an antibody chemotherapy combination. A remission rate in the range of 75-85% could compete with previous standard therapies.

The study was powered to detect with probability 95% an improvement of 20% points in overall response rate (ORR) to 85% for rituximab, gemcitabine and oxaliplatin compared to an ORR of ≤ 65% (null hypothesis). Therefore, a one-sided binomial test with a significance level of 0.05 was performed to test the primary outcome.

To evaluate the MTD, a minimum of 9–24 patients needed to be enrolled to the phase I part of the study. According to the primary objective of the phase II trial, the null hypothesis should be rejected with a probability of 95% if the actual remission rate is 85%. Based on these considerations, 48 evaluable observations were required for the dose expansion cohort. Assuming a drop-out rate of 10% (e.g. change in histology, treatment not started), it was planned to enroll 53 patients to the phase II part.

It was planned to prematurely terminate enrollment as soon as more than 10 patients of the planned evaluable 48 patients in the phase II part failed to achieve a remission as the null hypothesis cannot be rejected any more at this point.

All secondary endpoints were analyzed in a descriptive manner, numeric estimates were reported with two-sided 95% confidence intervals (CI). Time-to-event variables were described by Kaplan-Meier-estimates.

Cut-off date was 16.10.2015, the latest available date for medically reviewed study data.

## Results

### Patient characteristics

Between March 2004 and June 2009, the study enrolled 55 patients at 21 centers in Germany, 14 in the phase I and 41 in the phase II part.

**Fig. 1:**
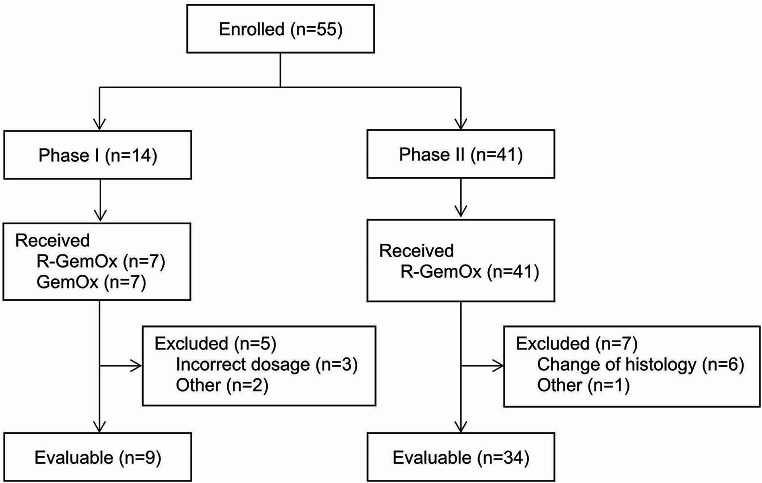
Primary analysis population

In the phase I trial period, 13 of 14 patients enrolled have received the full 4 cycles of treatment. Nine patients were evaluable for primary analysis (Fig. 1).

A total of 41 patients were enrolled in the phase II trial period, whereof 34 remained evaluable for primary analysis (Fig. 1). Twenty-nine patients (71%) received all 4 treatment cycles.

Clinical characteristics of evaluable patients are shown in Table 1. The most common histological subtypes were FL (*n* = 18) and MCL (*n* = 14). Patients received a median of 2 prior lines of therapy. Baseline characteristics of enrolled patients are listed in supplemental Table 4.

**Table 1:** Clinical characteristics of Evaluable Patients

Characteristic	Phase I population (*n* = 9) No (%)	Phase II population (*n* = 34) No (%)	Total population (*n* = 43) No (%)
Age (years) Median Range	71 56–79	68 31–82	69 31–82
Sex Male Female	3 (33) 6 (67)	17 (50) 17 (50)	20 (47) 23 (53)
ECOG 0–1 2 3	7 (78) 2 (22)	26 (76) 7(21) 1 (3)	33 (77) 9 (21) 1 (2)
Histological subtype FL grade 1/2/3A FL grade 3B FL NOS MCL Other B-NHLOther	3 (33) 1 (11) - 2 (22) 3 (33)	15 (44) - 2 (6) 12 (35) 5 (15)	18 (42) 1 (2) 2 (5) 14 (33) 8 (19)
Stage III IV	4 (44) 5 (56)	7 (21) 27 (79)	11 (26) 32 (74)
B-symptoms	2 (22)	14 (41)	16 (37)
Bone marrow involvement	4 (50); *n* = 8	20 (67); *n* = 30	24 (63); *n* = 38
Hemoglobin < 12 g/dl	4 (44)	15 (44)	19 (44)
LDH (> ULN)	6 (67)	20 (59)	26 (60)
R-GemOx	6 (67)	34 (100)	40 (93)
Previous treatment regimens Median Range	2 1–5	2 1–10	2 1–10
Prior aPBSCT	1 (11)	2 (6)	3 (7)
Prior antibody therapy	5 (55)	27 (79)	32 (74)
Prior salvage therapy	2 (22)	0 (0)	2 (6)
Prior remission	7 (78)	27 (82)	34 (79)

ECOG: Eastern Cooperative Oncology Group Performance Status

FL: Follicular lymphoma

MCL: Mantle cell lymphoma

B-NHL: B-cell non-Hodgkin’s lymphoma

LDH: Lactate dehydrogenase

NOS: Not otherwise specified

ULN: upper limit of normal

R-GemOx: Rituximab, gemcitabine and oxaliplatin

aPBSCT: autologous peripheral blood stem cell transplantation

### Primary outcome

#### Phase I - dose finding

In the phase I population, 7 patients were treated with GemOx and 7 with R-GemOx. Three patients at the first dose level of 70 mg/m^2^ and 6 patients at the second dose level of 80 mg/m^2^ were evaluable. No patient on dose level 1 received rituximab. None of the patients at the first dose level had a DLT. At the second dose level, three out of six patients had a DLT. In this group, all patients received rituximab as provided for by the protocol amendment. One patient developed hypotension, one neurotoxicity grade 3 and one had neutropenia as well as thrombocytopenia grade 4. Accordingly, dose level 1 was chosen for the phase II trial period.

### Efficacy

In the second study period, all patients received R-GemOx (*n* = 41). Twenty-eight patients (68%) completed all 4 cycles. In 9 patients (22%) a dose reduction was performed. After observing 11 patients who did not achieve a CR or PR, the recruitment was terminated prematurely in November 2009 for futility as per protocol because the null hypothesis could no longer be rejected (*p* = 0.45).

A total of 34 patients of 41 recruited patients were evaluable for the primary outcome analysis of the phase II cohort (Fig. 1).

Among 46 patients that were evaluable for the efficacy analysis, an overall response rate of 72% (33/46) was observed. One patient (2%) achieved CR and 32 patients PR (70%) (supplemental Table 2). Minimal remission (MR) and stable disease (SD) was observed in 4 patients (9%) and 5 patients (11%). Three patients (7%) progressed, and 1 patient (2%) died upon treatment with R-GemOx.

Among 22 patients with FL, ORR was 68% (14/22). Eleven out of 16 patients with MCL had CR or PR (ORR 69%).

### Secondary outcomes

The secondary intention-to treat (ITT) analysis of all recruited patients in the phase II population revealed an ORR of 64% (25/39, one-sided 95% CI: [50%; 100%]) (supplemental Table 5). Among all 55 recruited patients, ORR was 65% (31/48).

**Fig. 2:**
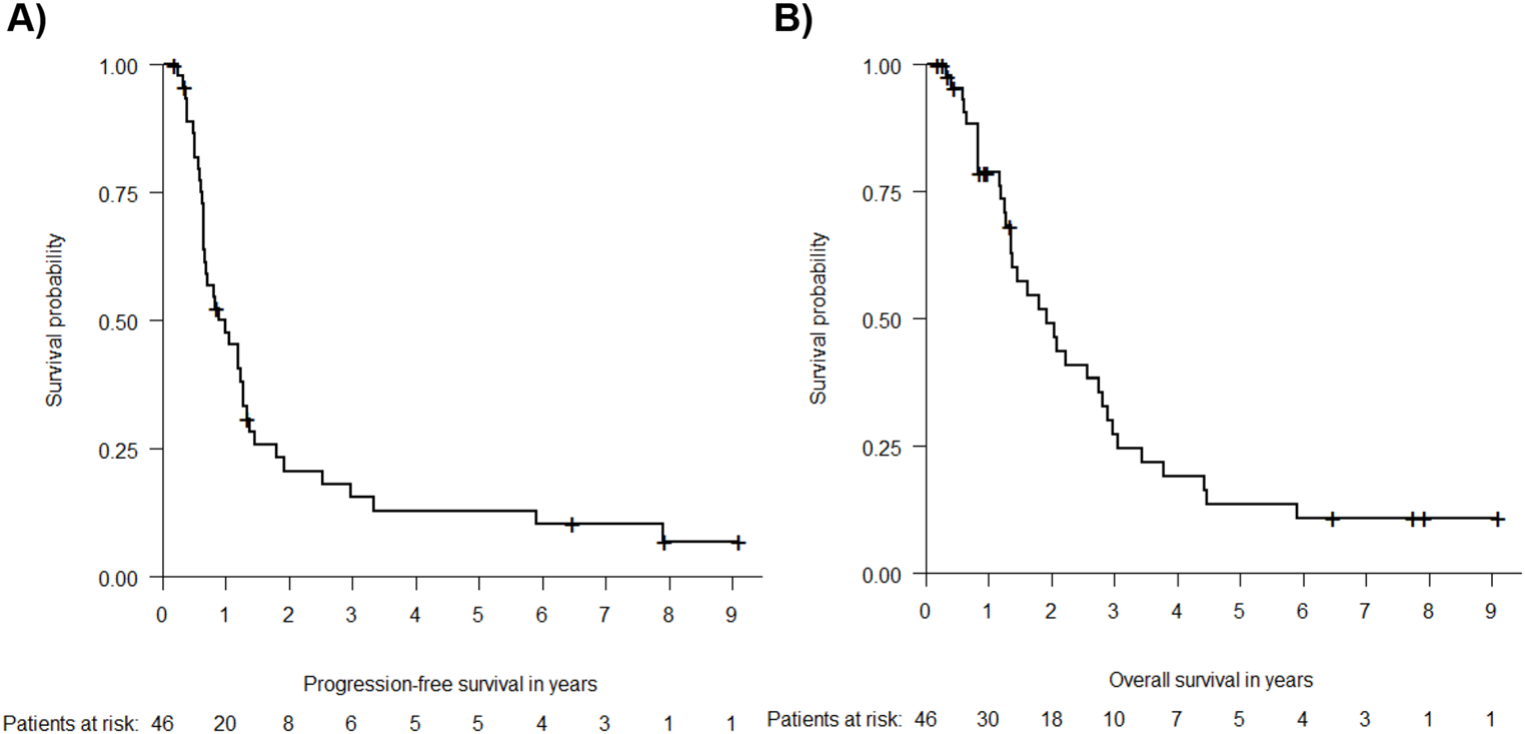
Progression-free (**A**) and Overall Survival (**B**) of evaluable patients (*n* = 46*). Kaplan-Meier estimates of PFS (**A**) and OS (**B**) among patients evaluable for the efficacy analysis from the phase I and II population. Censoring is indicated by crosses *n = 3 patients from the phase I population that were excluded for the dose finding analysis due to incorrect dosage were included for the efficacy analysis

After a median follow-up of 7.9 years and 7.7 years, median PFS of all evaluable patients (*n* = 46) was 1.0 years (Fig. 2A) and median OS was 2.1 years (Fig. 2B). Median PFS and OS of all 55 recruited patients was 0.8 and 1.9 years (supplemental Fig. 4).

**Fig. 3:**
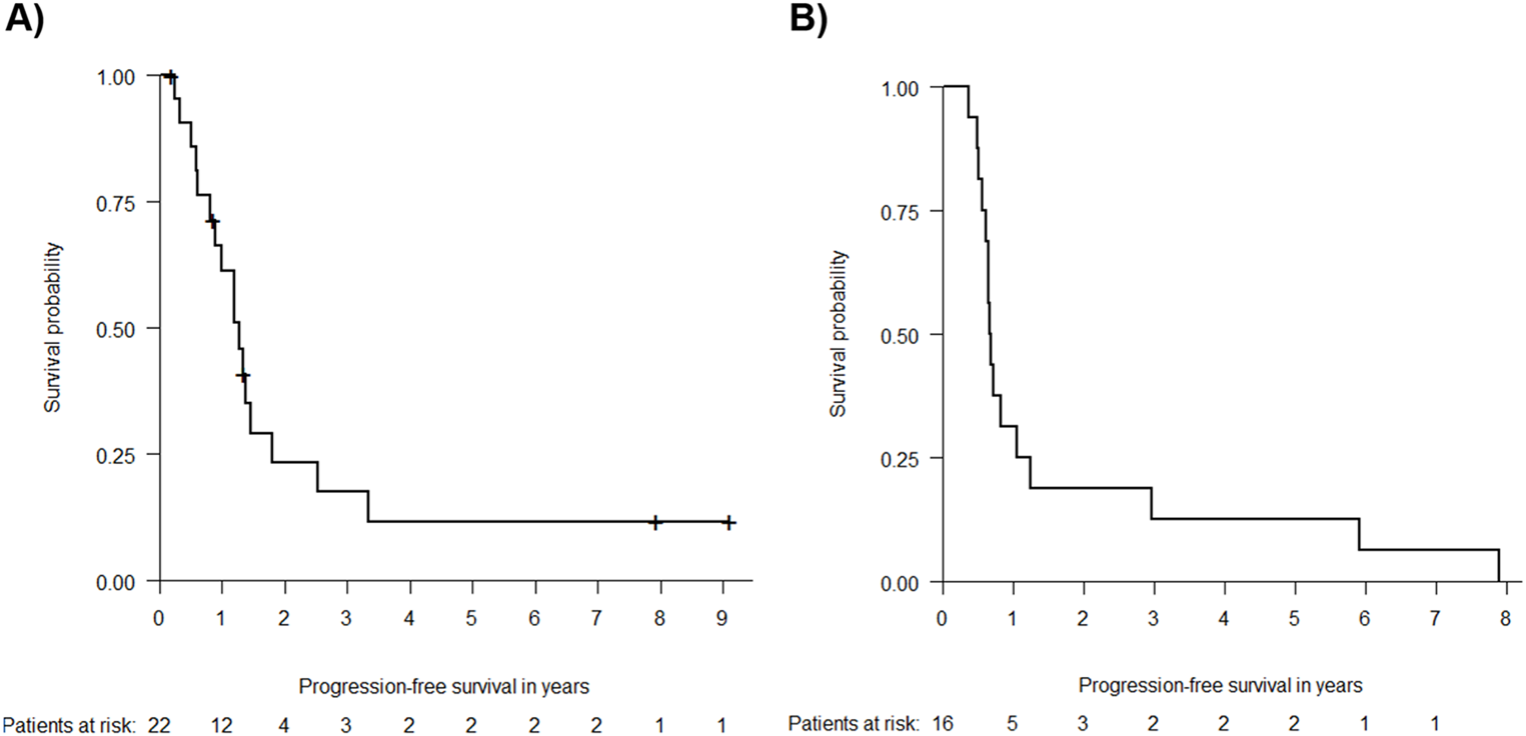
Progression-free survival of Follicular Lymphoma (**A**) (*n* = 22*) and Mantle Cell Lymphoma patients (**B**) (*n* = 16*) among evaluable patients (*n* = 46*) Kaplan-Meier estimates of PFS among patients with Follicular Lymphoma (**A**) and Mantle Cell Lymphoma (**B**). Censoring is indicated by crosses. *n = 3 patients from the phase I population that were excluded for the dose finding analysis due to incorrect dosage were included for the efficacy analysis

Median PFS and OS in FL patients was 1.3 (Fig. 3A) and 2.1 years, while MCL patients had a shorter PFS and OS of 0.7 (Fig. 3B) and 1.5 years.

### Safety

Overall, hematological toxicity was the most frequent grade ≥ 3 adverse event (AE) (supplemental Table 3). Among patients evaluable for safety analysis (*n* = 43), grade ≥ 3 leukopenia was observed in 19 (44%), granulocytopenia in 17 (40%), thrombocytopenia in 16 (37%) and anemia in 10 (23%). Peripheral neurotoxicity grade ≥ 3 was reported in 2 (5%) patients. Grade 3 or 4 fever, infection, nausea and vomiting, and mucositis only occurred in 1 patient (2%) each. There was no treatment-related death.

## Discussion

This single-arm, multicenter phase I/II clinical trial evaluated the maximum tolerated dose of oxaliplatin in combination with (rituximab-)gemcitabine and subsequently the efficacy of R-GemOx in recurrent or refractory MCL and indolent NHL. In this study, 70 mg/m^2^ of oxaliplatin was determined as MTD for the further conduct of the study. Remarkably, significantly higher doses of oxaliplatin ranging from 100 to 120 mg/m^2^ were applied in several published phase II studies investigating R-GemOx in lymphoma patients [34–36, 38]. However, at least in some of these trials G-CSF has been added to the regimen. In our study, the use of G-CSF was only optional. Despite the higher dosage, in studies that applied R-GemOx in the relapse setting, neutropenia grade ≥ 3 (47–73%), thrombocytopenia grade ≥ 3 (17–44%) and neurotoxicity grade ≥ 3 (0–8%) was comparable with the reported toxicity profile in our trial [34, 36, 38].

With an ORR of 72% the primary aim of the study was missed. Corazzelli and colleagues reported an ORR of 78% for R-GemOx in a study population that also included patients with DLBCL besides indolent lymphoma [38]. The response rate was higher in aggressive (79%) compared to indolent lymphoma (70%) (without MCL) [38]. Similar results were observed in another trial investigating R-GemOx in a mixed cohort of aggressive and indolent B-cell lymphoma with an ORR of 83% [34]. Notably, the addition of rituximab has substantially improved the response rate in the relapsed B-cell lymphoma [24, 38]. The disclosure of this data during the conduct of the study had prompted us to incorporate rituximab in this trial, nowadays being the reference treatment alongside a chemotherapy backbone.

The PFS of around 1 year is consistent with the results from the R-FCM trial without maintenance therapy [24]. In contrast, bendamustin-rituximab (BR) has been proven to be more effective [39].

Particularly in MCL, small subgroup analyses indicated decent response rates to R-GemOx [34, 38]. In our study, 69% of 16 MCL patients achieved PR or CR. However, as novel substances like ibrutinib have proven to be superior to conventional chemotherapy in early relapses of MCL, R-GemOx might be an alternative option for later relapses occurring after > 24 months [10]. In the era of cellular therapy, it might be used as bridging to CAR-T cell therapy.

In r/r FL, several novel treatments such as rituximab-lenalidomide, mosunetuzumab or CAR-T-cells have recently been approved, making R-GemOx obsolete in this indication [21, 40, 41].

Hematological toxicity constitutes by far the most common grade 3/4 adverse events in this trial, being in line with previously reported trials evaluating GemOx in r/r B-cell lymphoma patients [34, 36, 38]. However, except for one grade 4 infection no cases of severe infections or bleeding were reported.

Interestingly, when R-GemOx was applied in a first-line setting, hematotoxicity was significantly lower suggesting limited bone marrow reserve poses an important risk for developing severe cytopenias [35]. Comparable toxicities were observed with other relapse regimens such as R-FCM and BMR [24, 42]. In contrast, BR offers a more favorable safety profile [39].

Peripheral neurotoxicity grade ≥ 3 occurred in 5% of the patients corresponding to the results in previously published trials with oxaliplatin and gemcitabine [35, 36, 38].

In conclusion, the study failed to meet its primary aim, though it could be demonstrated that the combination of rituximab, gemcitabine with oxaliplatin at a dose of 70 mg/m^2^ is safe and feasible in r/r indolent and mantle cell lymphoma. Although the reported ORR of 72% demonstrates a significant anti-lymphoma efficacy, other combinations such as BR offer a more favorable efficacy and safety profile [39]. The approval of bispecific antibodies, immunomodulatory drugs and CAR-T-cells in recent years has displaced the use of conventional chemotherapy to a large part in the therapeutic landscape of r/r indolent and mantle cell lymphoma. In our opinion, R-GemOx only represents an alternative option in late relapses or for bridging to CAR-T-cell therapy specifically in MCL.

### Electronic supplementary material

Below is the link to the electronic supplementary material.

Supplementary Material 1
