# Supplementary material for: Rituximab, gemcitabine and oxaliplatin in relapsed or refractory indolent and mantle cell lymphoma: results of a multicenter phase I/II-study of the German Low Grade Lymphoma Study Group
Source: Ann Hematol. 2024 Mar 9;103(7):2373–80. doi: 10.1007/s00277-024-05689-w (PMC11224115; doi:10.1007/s00277-024-05689-w)
Supplement: Supplementary file 1 — Supplementary Material 1 [file 277_2024_5689_MOESM1_ESM.docx]

**SUPPLEMENT**

***Supplemental Methods***

Definition of response criteria

Complete remission (CR)

Complete regression of all objective disease findings at the time of restaging with complete regression of all pre-existing lymph node involvements, hepatomegaly or splenomegaly for at least four weeks. Exclusion of bone marrow infiltration by biopsy and normalization of all certain lymphoma-associated laboratory parameters.

Partial remission (PR)

At least a 50% decrease of all measurable and evaluable lymphoma manifestations (sum of the product of the diameters (SPD) measured in two perpendicular dimensions) for at least four weeks without occurrence of new lesions.

Minimal response (MR)

Decrease of all measurable lymphoma manifestations by less than 50%.

Stable disease (SD)

No regression of the measurable lymphoma manifestations.

Progressive disease (PD)

Increase of frequency and severity of disease-specific symptoms or occurrence of any new nodal or extranodal lymphoma manifestations or at least a 25% increase of SPD of pre-existing lymphoma manifestations or at least a 25% increase of splenomegaly.

Inclusion criteria

- Patients with relapsed or refractory indolent NHL requiring treatment
- Histologically confirmed disease (WHO classification: Follicular lymphoma grade I or II, mantle cell lymphoma, lymphoplasmacytic lymphoma (immunocytoma), nodal or splenic marginal zone lymphoma, lymphocytic lymphoma with plasmocytic differentiation)
- Measurable tumor lesion
- No cytostatic therapy during the last 4 weeks before study begin
- ECOG performance-status 0-2
- Estimated life-expectancy of at least 12 weeks
- Age ≥ 18 years
- Written informed consent

Exclusion criteria

- Patient who are eligible for high-dose therapy
- Secondary high malignant lymphoma
- Prior malignancies as far as cure cannot be assumed according to the current knowledge
- Severe general disorder (according to the assessment of the investigator)
- Non-lymphoma related hepatopathy with transaminase levels > 3 x ULN and/or bilirubin levels > 2.0 mg/dl
- Non-lymphoma related renal failure (serum creatinine > 2.0 mg/dl)
- Pregnant or lactating women
- Absolute neutrophil count (ANC) < 1.5/nl or platelets < 100/nl if not caused by bone marrow involvement
- Known central nervous system disease (e.g. metastases, treatment-refractory seizure disorder)
- Severe psychiatric disease
- HIV-infection
- Acute infections that cannot be controlled
- Allergies to murine protein

***Supplemental Tables***

**Supplemental Table 2: Responses of Evaluable Patients after (R)-GemOx treatment**

| **Response** | **Phase I population* (n=12)**  **No (%)** | **Phase II population (n=34)**  **No (%)** | **Total population***  **(n=46)**  **No (%)** | **MCL population***  **(n=16)**  **No (%)** | **FL population***  **(n=22)**  **No (%)** |
| --- | --- | --- | --- | --- | --- |
| CR | - | 1 (3) | 1 (2) | - | 1 (5) |
| PR | 8 (67) | 24 (71) | 32 (70) | 11 (69) | 14 (64) |
| MR | 1 (8) | 3 (9) | 4 (9) | 3 (19) | 1 (5) |
| SD | 2 (17) | 3 (9) | 5 (11) | 2 (13) | 3 (14) |
| PD | 1 (8) | 2 (6) | 3 (7) | - | 2 (9) |
| ED | - | 1 (3) | 1 (2) | - | 1 (5) |
| CR/PR | 8 (67) | 25 (74) | 33 (72) | 11 (69) | 15 (68) |

*n=3 patients from the phase I population that were excluded for the dose finding analysis due to incorrect dosage were included for the efficacy analysis

CR, complete remission; PR, partial remission; MR, minimal remission; SD, stable disease; PD, progressive disease; ED, early death

**Supplemental Table 3: Adverse Events^†^ of Evaluable Patients per patient (maximum occurred grade)**

| **Any AE** | **Phase I population Dose level 1 (n=3) No (%)** | | | **Phase I population Dose level 2 (n=6) No (%)** | | | **Phase II population**  **(n=34) No (%)** | | |
| --- | --- | --- | --- | --- | --- | --- | --- | --- | --- |
|  | Grade 1-2 | Grade 3 | Grade 4 | Grade 1-2 | Grade 3 | Grade 4 | Grade 1-2 | Grade 3 | Grade 4 |
| Hemoglobin | 2 (67) | - | - | 4 (67) | - | 1 (17) | 16 (47) | 7 (21) | 2 (6) |
| Thrombocytes | 1 (33) | - | - | 2 (33) | 1 (17) | 3 (50) | 6 (18) | 7 (21) | 5 (15) |
| Leukocytes | 1 (33) | - | - | 2 (33) | 2 (33) | 2 (33) | 13 (38) | 12 (34) | 3 (9) |
| Neutrophils | 1 (33) | - | - | 1 (17) | - | 4 (67) | 10 (29) | 5 (15) | 8 (24) |
| Hemorrhage | - | - | - | 1 (17) | - | - | 2 (6) | - | - |
| Fever | 2 (67) | - | - | 1 (17) | 1 (17) | - | 7 (21) | - | - |
| Infection | - | - | - | - | - | 1 (17) | 4 (12) | - | - |
| Alopecia | - | - | - | 1 (17) | - | - | 9 (26) | - | - |
| Nausea/Vomiting | 1 (33) | - | - | 1 (17) | - | - | 16 (47) | 1 (3) | - |
| Mucositis | - | - | - | - | - | - | - | 1 (3) | - |
| Obstipation | 1 (33) | - | - | - | - | - | 5 (15) | - | - |
| Diarrhea | - | - | - | - | - | - | 6 (18) | - | - |
| Cardiac function | - | - | - | 1 (17) | - | - | 2 (6) | - | - |
| Arrhythmia | - | - | - | - | - | - | 4 (12) | - | - |
| Hepatic toxicity | 2 (67) | - | - | - | - | - | 5 (15) | - | - |
| Peripheral neurotoxicity | 3 (100) | - | - | 3 (50) | 1 (17) | - | 12 (34) | 1 (3) | - |

† According to WHO Handbook for reporting results of cancer treatment, No. 48 (1979), WHO Offset Publications, Geneva

**Supplemental Table 4: Clinical characteristics of Enrolled Patients**

| **Characteristic** | **Phase I population (n=14)**  **No (%)** | **Phase II population (n=41)**  **No (%)** | |
| --- | --- | --- | --- |
| Age (years)  Median  Range | 71  55-79 | 69  31-82 | |
| Sex  Male  Female | 6 (43)  8 (57) | 21 (51)  20 (49) | |
| ECOG  0-1  2 | 11 (79)  3 (21) | 32 (78)  9 (22) |  |
| Histological subtype  FL grade 1/2  FL grade 3A  FL grade 3B*  FL NOS  MCL  Lymphoplasmacytic lymphoma  Hodgkin’s lymphoma, mixed cellularity, NOS  B-CLL*  DLBCL*  Splenic MZL  MZL, NOS | 4 (29)  0 (0)  1 (7)  1 (7)  5 (36)  2 (14)  1 (7)  0 (0)  0 (0)  0 (0)  0 (0) | 14 (34)  1 (2)  1 (2)  3 (7)  12 (29)  1 (2)  0 (0)  4 (10)  1 (2)  2 (5)  2 (5) | |
| Stage  III  IV | 6 (43)  8 (57) | 8 (20)  33 (80) | |
| B-symptoms | 2 (14) | 14 (34) | |
| Bone marrow involvement | 6 (50); n=12 | 24 (67); n=36 | |
| Hemoglobin <12 g/dl | 8 (57) | 19 (46) | |
| LDH (>ULN) | 8 (57) | 23 (56) | |
| R-GEMOX | 7 (50) | 41 (100) | |
| Previous treatment regimens  Median  Range | 2  1-4 | 2  1-10 | |
| Prior aPBSCT | 1 (7) | 3 (7) | |
| Prior antibody therapy | 10 (71) | 33 (80) | |
| Prior salvage therapy | 2 (14) | 0 (0) | |
| Prior remission | 9 (64) | 29 (74) | |

*excluded

ECOG: Eastern Cooperative Oncology Group Performance Status

LDH: Lactate dehydrogenase

NOS: Not otherwise specified

ULN: upper limit of normal

R-GemOx: Rituximab, gemcitabine and oxaliplatin

aPBSCT: autologous peripheral blood stem cell transplantation

**Supplemental Table 5:**

**Responses of Enrolled Patients after (R)-GemOx treatment (secondary intention-to-treat analysis)**

| **Response** | **Phase I population Dose level 1 (n=6)**  **No (%)** | **Phase I population Dose level 2 (n=8)**  **No (%)** | **Phase II population (n=41)**  **No (%)** |
| --- | --- | --- | --- |
| CR | - | - | 1 (2) |
| PR | 2 (33) | 4 (50) | 24 (59) |
| MR | - | 1 (13) | 3 (7) |
| SD | 1 (17) | - | 4 (10) |
| PD | - | 1 (13) | 6 (15) |
| ED | - |  | 1 (2) |
| NE | 3 (50) | 2 (25) | 2 (5) |
| CR/PR (available staging) | 2 (67) | 4 (67) | 25 (64) |

CR, complete remission; PR, partial remission; MR, minimal remission; SD, stable disease; PD, progressive disease; ED, early death; NE, not evaluable. CR/PR of patients with available staging result.

***Supplemental Figures***

**A) B)**

**
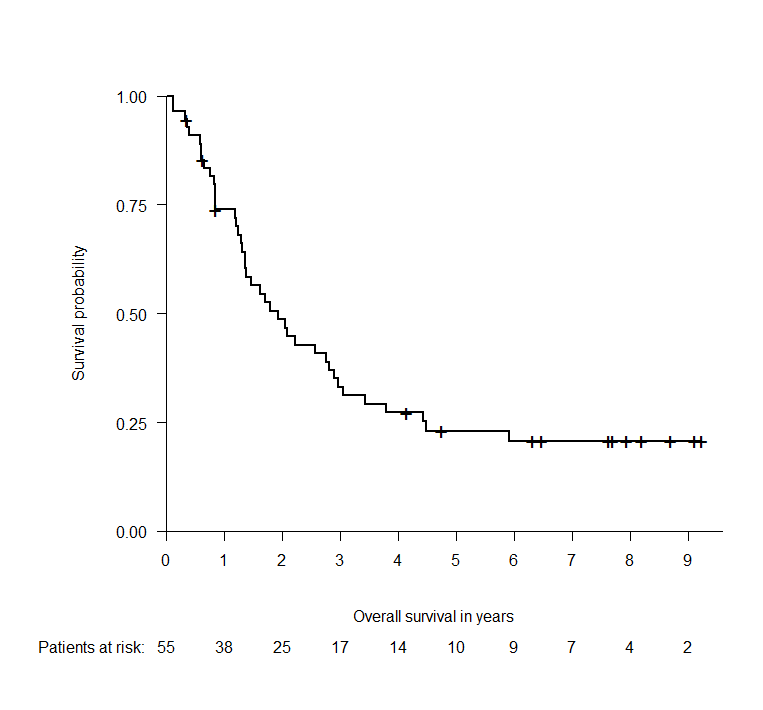
**
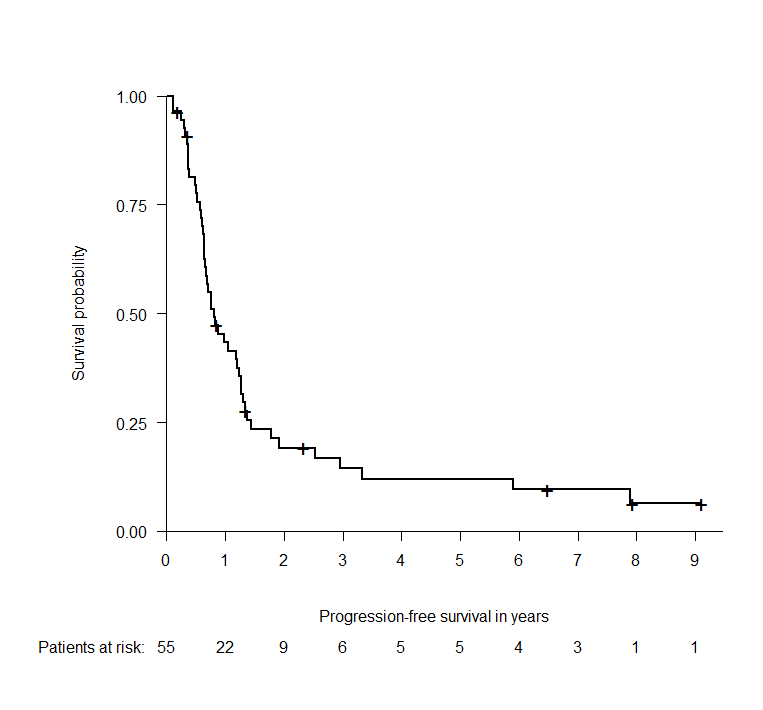


**Supplemental Fig. 4: Progression free (A) and Overall Survival (B) of all enrolled patients.**

Kaplan-Meier estimates of PFS (A) and OS (B) among patients that were enrolled to the study. Censoring is indicated by crosses.

***NHL 2003 09-03 GO Trial Investigators***

| Center | Investigators |
| --- | --- |
| St. Marien-Hospital Hagen, Germany | Dr. Hans-Walter Lindemann, Cordula Maciejewski, Maria Liliana Garbarino Gonzales |
| Medical Clinic A, Clinical Centre Ludwigshafen, Ludwigshafen, Germany | Prof. Dr. Michael Uppenkamp, Dr. Martin Hoffmann, Dr. Burkhard Weiß |
| Hematology-Oncology Practice, Offenbach, Germany | Dr. Hans-Peter Böck, Dr. Harald E. Ballo |
| Hematology-Oncolotgy, Caritas Hospital, Lebach, Germany | Dr. Stephan Kremers, PD Dr. Stefan Bauer, Dr. Gero Leonhard-Helmschmidt |
| University Hospital, University of Duisburg-Essen, Essen, Germany | Prof. Dr. Ulrich Dührsen, Prof. Dr. Jan Dürig |
| Hematology-Oncology, St. Georg Clinic, Leipzig, Germany | Dr. Luisa Mantovani Löffler, Dr. Christoph Schimmelpfennig, Dr. Albrecht Kretzschmar |
| Internal Medicine, Diakonieklinikum, Stuttgart, Germany | Prof. Dr. Jochen Greiner, Dr. Rudolf Mück, Dr. Joachim Kaesberger |
| Hematology-Oncology Practice, Bad Soden, Germany | Dr. Gernot Seipelt, Dr. Ursula Koch |
| Hematology-Oncology Practice, Landshut, Germany | Dr. Ursula Vehling-Kaiser, Dr. Friedhelm Woitinas, Dr. Franziskus Finterwalder |
| Medical Clinic II, Hematology, Oncology, Schwarzwald-Baar Clinic, Villingen-Schwenningen, Germany | Prof. Dr. Wolfram Brugger, Dr. Friedemann Köhler, Dr. Christof Burkhart |
| Medical Clinic I, Hematology, Oncology, Vinzentius hospital, Landau, Germany | Dr. Matthias Kraft, Dr. Martin Schröder, Dr. Martina Varrentrapp |
| Medical Clinic II, Hematology, Oncology, community hospital Braunschweig, Braunschweig, Germany | Prof. Dr. Jürgen Krauter, Dr. Konrad Namberger, Dr. Miriam Ahlborn |
| Sana Hospital Hof, Oncology, Hematology, Hof, Germany | Prof. Dr. Friedrich-W. Busch, Dr. Constanze Lohse, Dr. Constanze Weiner |
| Hematology-Oncology Practice, Ansbach, Germany | Dr. Markus Hahn, Dr. Sebastian Müller |
| Department of Internal Medicine III, LMU University Hospital Munich, Munich, Germany | Prof. Dr. Wolfgang Hiddemann, Prof. Dr. Martin Dreyling, Dr. Roswitha Forstpointner |
| Medical Clinic I, Westpfalz Hospital Kaiserslautern, Kaiserslautern, Germany | Prof. Dr. Hartmut Link, Dr. Stefan Mahlmann, Dr. Sven Lichtenberger |
| Medical Clinic I, Kliniken Maria Hilf GmbH, Mönchengladbach, Germany | PD Dr. Ullrich Graeven |
| Department for Internal Medicine, Marien-Hospital Bottrop, Bottrop, Germany | Dr. Friedrich Schorr, PD Dr. Klaus Mönkemüller |
| Medical Clinic III, Klinikum Südstadt, Rostock, Germany | Dr. Beate Krammer-Steiner, Dr. Kerstin Peters, Anke Lohse |
| Hematology, Oncology, Asklepios Clinic St. Georg, Hamburg, Germany | Prof. Dr. Norbert Schmitz, Dr. Maike Nickelsen |
| Medical Clinic II, St. Johannes-Hospital, Dortmund, Germany | PD Dr. Ralf Georg Meyer, Dr. Volker Hagen |
